# Distribution of Opportunistic Pathogens in People Living with HIV at a University Hospital in Istanbul over a One-Year Treatment Period and Its Association with CD4 T Cell Counts

**DOI:** 10.3390/pathogens12101226

**Published:** 2023-10-09

**Authors:** Hayriye Kirkoyun Uysal, Muammer Osman Koksal, Kutay Sarsar, Pinar Soguksu, Gonca Erkose Genc, Gizem Yapar, Evrim Ozdemir, Mustafa Onel, Sevim Mese, Mehmet Demirci, Zayre Erturan, Eray Yurtseven, Omer Haluk Eraksoy, Ali Agacfidan

**Affiliations:** 1 Department of Medical Microbiology, Istanbul Faculty of Medicine, Istanbul University, Istanbul 34093, Turkeyevrimozdemir2020@ogr.iu.edu.tr (E.O.); onelm@istanbul.edu.tr (M.O.); ali.agacfidan@istanbul.edu.tr (A.A.); 2 Department of Medical Microbiology, Faculty of Medicine, Kırklareli University, Kırklareli 39100, Turkey; 3 Department of Biostatistics, Istanbul Faculty of Medicine, Istanbul University, Istanbul 34093, Turkey; eyurt@istanbul.edu.tr; 4 Department of Infectious Diseases and Clinical Microbiology, Istanbul Faculty of Medicine, Istanbul University, Istanbul 34093, Turkey

**Keywords:** HIV, CD4/CD8 ratio, opportunistic infections, CMV, EBV

## Abstract

Among sexually transmitted diseases, HIV causes very serious clinical manifestations that can lead to death. As a result, millions of people have to live with this problem that threatens their health. The virus attacks the immune system of the host, especially CD4+ T lymphocytes, causing the suppression of the immune system. CD4, CD8 counts, and HIV RNA viral loads are monitored in HIV-infected patients with antiretroviral treatment, and CD4 counts play an important role in determining the effectiveness of the treatment. Despite the advances in treatment in the present day, opportunistic infections are the main cause of morbidity and mortality in these patients, and the evaluation of immunological parameters is valuable for the prognosis of the disease in this process. In the present study, the purpose was to investigate the opportunistic infections faced by naive HIV-positive patients who applied to our laboratory and were diagnosed between 2019 and 2022 during their one-year treatment period, and the correlation of the immunological parameters was also evaluated retrospectively using the hospital automation system and laboratory data. A total of 107 opportunistic causative microorganisms were identified in 87 of the 230 HIV-positive patients over one year. *T. pallidum* was detected in 43 (18.6%) of these patients, Cytomegalovirus (CMV) in 32 (13.9%), Epstein–Barr virus (EBV) in 9 (3.9%), Hepatitis B virus (HBV) in 10 (4.3%), *C. albicans* in 7 (3%), *M. tuberculosis* in 3 (1.3%), Hepatitis C virus (HCV) in 2 (0.8%), and *C. glabrata* in 1 (0.4%) patient. Although mono-agent co-infections were determined in 69 of 87 people living with HIV, two-agent co-infections were detected in 16 HIV patients, and three-agent co-infections were identified in two HIV patients. Considering the correlation between the CD4/CD8 ratio and infection positivity, a moderate negative correlation was determined with HIV RNA viral load and CMV infection. The CD4/CD8 ratio had a low negative correlation with EBV and C. albicans infections. It was also found that the follow-up of HIV RNA load in the diagnosis of *T. pallidum*, CMV, EBV, and *C. albicans* may be meaningful. Opportunistic infections mainly affect immunosuppressed patients and can be prevented with effective treatment. Although it is already known that HIV patients may face different infections during their treatment, it was concluded that more attention should be paid to *T. pallidum*, CMV, EBV, and C. albicans agents. These infections should be routinely monitored with HIV viral load and the CD4/CD8 ratio.

## 1. Introduction

Opportunistic infections are common in Human Immunodeficiency Virus (HIV) infection and usually appear as the reactivation of a latent infection or infections caused by newly acquired low-virulence microorganisms. The development of certain strategies, the application of chemoprophylaxis, and immunization can improve the survival and quality of life of individuals. The incidence of such infections (and hence morbidity and mortality) is reduced with the use of effective Antiretroviral Treatment (ART) regimens. Providing viral suppression can eliminate most of the opportunistic factors [1,2]. However, opportunistic infections continue to be detected because HIV infections cannot be diagnosed, people are under severe immunodeficiency when positivity is detected, and permanent viral suppression cannot be achieved when diagnosed patients do not receive regular HIV care or ART [3,4].

HIV infection shows a wide spectrum, from asymptomatic carriers to severe clinical manifestations accompanied by opportunistic infections. Infections and malignancies may occur in untreated cases, and Acquired Immunodeficiency Syndrome (AIDS) may develop. Also, the virus escapes from the immune system and creates a chronic persistent infection affecting the immune system at various stages. The most important among these mechanisms is the emergence of mutations and recombinations, resulting in viral diversity, with a high-speed viral replication level. Essentially, the main mechanism impairing the immune system is the depletion (immunodeficiency) in CD4 T cells and immune system activation [5,6]. The data obtained from the CDC indicate that outpatients cause a decrease in the incidence of hospitalization and mortality. Delayed admissions can be a cause of mortality. Cohort studies of HIV-seropositive individuals show that opportunistic factors continue to be the most common cause of mortality [7,8].

Many previous studies have shown that CD4 T lymphocytes are highly associated with opportunistic infections, and the incidence can be variable in different geographical areas [9,10]. It is known that pathogens such as CMV and EBV can reactivate during HIV infection, especially when CD4 counts decrease [10]. In the present day, early diagnosis, regular follow-up, and ART can prevent disease progression and reduce morbidity and mortality. However, the right approach, initial evaluation, and regular follow-up are extremely important to achieve these targets [9,10]. In the present study, the purpose was to investigate the opportunistic infections faced by naive HIV-positive patients who applied to our laboratory and were diagnosed between 2019 and 2022 during their one-year treatment period. In this regard, the co-infections of our HIV-positive patients during this period and the correlation of their immunological parameters were also evaluated.

## 2. Materials and Methods

People who applied to Istanbul Medical Faculty, Infectious Diseases, and Clinical Microbiology Department Clinic with a suspicion of HIV were examined clinically, and the diagnostic tests were performed in the laboratories of the Medical Microbiology Department. In the present study, the infections faced by HIV patients who were diagnosed between 2019 and 2022 in a one-year treatment period were examined. Opportunistic infections faced by naive HIV-positive patients who applied to our laboratory and were diagnosed between 2019 and 2022 during their one-year treatment period were evaluated retrospectively using the hospital automation system and laboratory data. Patients were evaluated at time intervals.

ELISA tests are fast, reliable, and economical for HIV diagnosis. Antibody tests may be negative during the window period when active replication is detected and the contagiousness is high because of high viremia. The probability of false negatives is greatly reduced with the 4th-generation ELISA tests that detect antigens and antibodies together. Anti-HIV 1/2 antibodies were routinely investigated in our laboratory using two different 4th-generation ELISA kits (Genscreen Ultra HIV Ag-Ab Bio-Rad France (Marnes-la-Coquette, France), Vironostika HIV Ag/Ab Biomerieux France (Craponne, France)). The western blot method was used to confirm positive tests (INNO-LIA HIV I/II Score FUJİREBİO).

The follow-up of the patients continued with the HIV RNA viral load. Viral load detection was achieved using a QIAsymphony SP/AS and Rotor-Gene Q (QS-RGQ System) (Hilden, Germany) device with an Artus™ HIVirus-1 kit. The Artus™ HI Virus-1 RG RT-PCR kit is an in vitro nucleic acid amplification assay used for the quantification of HIV type-1 (HIV-1) RNA in human plasma. This diagnostic test kit employs Reverse Transcription Polymerase Chain Reaction (RT-PCR). The test can quantify HIV-1 RNA within the range of 120 − 1 × 10^8^ HIV-1 IU/mL.

The TPHA and RPR tests were used for the diagnosis of Treponema pallidum. Similarly to HIV, the diagnosis of CMV and EBV was achieved in the Virology Laboratory using a Rotor-Gene Q Real-Time PCR System (Hilden, Germany). Serological markers and molecular methods were used for the patients in the Virology Laboratory for the diagnosis of HAV, HBV, and HCV. The identification was provided in the Mycobacteriology Laboratory using Bactec MGIT960 and Löwenstein–Jensen broth media for the diagnosis of M. tuberculosis. Chromagar-Candida (Chromagar, France) and Sabouraud dextrose agar were used in the Mycology Laboratory for the diagnosis of *Candida* spp.

The immunological parameters of the patients were tested using a flow cytometry device, and the results were evaluated. CD4 (helper T cells) were prepared using monoclonal antibody CD45-FITC/CD4-RD1/CD8-ECD/CD3-PC5 (Beckman Coulter, Inc, Brea, CA, USA) to determine absolute and percent values and CD4/CD8 ratios of CD8 (cytotoxic T cells) with a lyse-wash protocol. CD4 and CD8 absolute values were obtained by adding flow-count fluorosphere solution to the samples. The evaluation of the samples was conducted via a flow cytometry device (Beckman Coulter Navios Ex) using NaviosEx software version 2.0.

SPSS version 25 software was used for statistical analysis; non-normally distributed data are given as median and IQR, and categorical data are given as numbers and percentages. The Mann–Whitney U test was used for the comparison of the two groups. Correlation analysis was performed using Spearman correlation analysis because the data did not show a normal distribution. In the evaluation of the correlation analysis, we took the following rules into account: low correlation = 0.1–0.29; moderate correlation = 0.30–0.49; high correlation = 0.50–1.00; negative values indicate negative correlations; and positive values indicate positive correlations. ROC curve analysis was used to find the CD4/CD8 ratio and HIV RNA cut-off values for infection positivity. The Spearman correlation was examined because the numerical variables did not have a normal distribution. The Chi-square test was used to correlate two categorical variables, and one-way ANOVA was used for the correlation of numerical–categorical variables.

## 3. Results

HIV patients diagnosed between 2019 and 2022 were examined in terms of opportunistic factors, and opportunistic infections were detected in 87 of 230 patients. Table 1 shows the characteristics of the patients included in the study.

The most common agents were *T. pallidum* in 43 (18.6%) patients, Cytomegalovirus in 32 (13.9%), Epstein–Barr virus in 9 (3.9%), *Candida albicans* in 7 (3%), Hepatitis B virus in 10 (4.3%), Hepatitis C virus in 2 (0.8%), and *M. tuberculosis* in 3 (1.3%) (Table 2).

The TPHA and RPR tests were used for the diagnosis of Treponema pallidum. The number of people who were positive for both tests was found to be 29. There were 12 patients with TPHA-positive RPR test results and 2 patients with TPHA-negative RPR-positive test results. In the present study, syphilis–HIV co-infections were determined as the most prevalent co-infections (Table 2). A total of 198 patients were examined for HBsAg, and HBV DNA positivity was detected in 10 patients with HBsAg positivity. Four patients were evaluated as Occult (anti-HBc IgG-positive, HBsAg-negative). Anti-HCV antibody positivity was detected in two patients. Co-infection with HBV and HCV was not detected in the patients. HAV-IgG positivity was detected in 40 patients, and HAV-IgM positivity was also detected in 1 patient.

Diagnosis was achieved in 32 CMV-positive patients and 9 EBV-positive patients by detecting virus DNA with Real-Time PCR. M. tuberculosis was detected in three patients as a respiratory-tract agent. The identification was achieved using Bactec MGIT960 and Löwenstein–Jensen broth media. Chromagar-Candida (Chromagar, Paris, France) and Sabouraud dextrose agar were used for the isolation of *Candida* spp. *Candida albicans* was produced from the sputum samples of three patients, broncholavage samples of two patients, and a throat swab. *C. albicans* and *C. glabrata* were isolated from the mouth swabs of one patient (Table 2).

Although mono-infectious agents were detected in 69 of 87 patients with opportunistic agents, two agents were detected in 16 patients, and three agents were detected in 2 patients. TPHA and CMV test positivity was detected in six patients with two agents, CMV and EBV positivity in four, HBV and TPHA positivity in two, and CMV and HBV positivity in two patients, while CMV + C. albicans and M. tuberculosis + C. albicans were found together in one patient, respectively. Triple co-infection was detected as EBV + CMV + C. albicans in one patient, while HCV + *C. glabrata* + *C. albicans* was detected in one patient. The infectious agents and distribution of CD4 numbers are given in Table 3.

**Figure 1:**
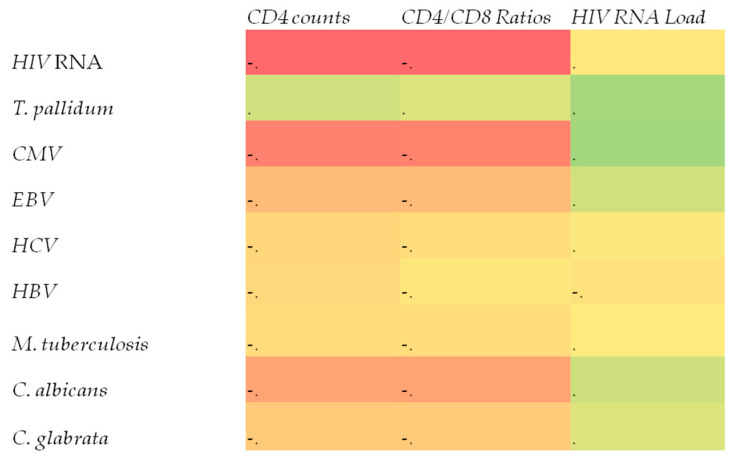
Correlations between the presence of these microorganisms and immune parameters (red: negative correlation, yellow: near zero, green: positive correlation).

The correlations between the CD4 count, CD4/CD8 ratio, HIV viral load, and infection positivity are given in Table 4 and Table 5. Considering the correlation between the CD4 count and infection, a moderate and negative correlation was detected with HIV RNA viral load, and a similar negative correlation was detected with CMV infection. Although the CD4 count showed a low and negative correlation with EBV and C. albicans infections, it was found to have a low level of positive correlation with T. pallidum infection. When the correlation between the CD4/CD8 ratio and infection positivity was evaluated, a moderate and negative correlation was detected with HIV RNA viral load, similar to CD4, and a moderate and negative correlation was detected with CMV infection. The CD4/CD8 ratio had a low and negative correlation with EBV and C. albicans infections. No correlation was detected with *T. pallidum* infection. Considering the correlation between HIV RNA viral load and infection positivity, a low and positive correlation was found with *T. pallidum*, CMV, EBV, and *C. albicans* infection positivity (Table 5 and Figure 1).

When the HIV viral load and a cut-off value that could be detected for the CD4/CD8 ratio regarding positivity for different infections were evaluated, Table 6 was obtained. HIV RNA load was significant for *T. pallidum*, CMV, EBV, and *C. albicans*.

## 4. Discussion

Opportunistic infections that are associated with HIV involve various life-threatening infections. The virus is a retrovirus attacking CD4+ T lymphocytes. When the number of T cells decreases, susceptibility to secondary infection develops. It is already known that HIV-1 is frequently detected all over the world. The evaluation and management of these infections, as well as the follow-up of effected patients, emphasize the role of an interprofessional team. For this reason, close follow-up must be performed to ensure regular treatment and prevent viral/bacterial/fungal diseases [11,12]. For this reason, we focused on the opportunistic infections faced by HIV patients and the correlation of these infections with the CD4/CD8 ratio and HIV viral load in the present study.

Viral replication can decrease to undetectable levels as a result of antiretroviral treatment during an HIV infection. However, it is already known that chronic persistent inflammation associated with the progression of the disease continues for life. Immune system functions are rearranged, and the incidence of opportunistic infections decreases with ART. Non-compliance with the treatment causes resistance to the drugs, an increase in HIV viral load, and a decrease in the number of CD4+ cells, preparing the ground for the development of opportunistic infections [13,14].

Syphilis continues to be a worldwide public healthcare concern, with an increasing incidence of people living with HIV/AIDS in recent years [15]. As a result of the effects of an HIV infection on the immune system, susceptibility to other Sexually Transmitted Infections (STIs) may increase, and people with weakened immunity may have a less protective response to sexually transmitted pathogens. Systematic studies conducted to evaluate the prevalence of STIs in people living with HIV/AIDS emphasize that syphilis is one of the most common infections in this group. Here, the researchers would like to underline that the rates of syphilis co-infection varied greatly in HIV-infected individuals in previous studies, with a range of 2–43% in Europe and 1–21% in North America [16]. In the present study, seroprevalence was detected as 43/230 (18.6%) for syphilis, which is compatible with the data of other studies. In another study that was conducted in our country, syphilis seroprevalence was investigated in 224 HIV-positive male patients in Istanbul, where HIV prevalence is high. Positivity was found in 47 patients (19.3%). The study showed that the frequency of syphilis is high in HIV-infected patients in Turkey, in agreement with other studies conducted in different parts of the world. However, it also emphasized that HIV-infected patients must be screened for syphilis at least once a year and informed about STDs. The measures taken have critical importance in terms of public healthcare to prevent HIV and syphilis [17].

The CDC recommends that HIV-positive patients be tested for Hepatitis B virus (HBV) because they share common transmission routes, which occur as a result of contact with the body fluids of infected patients and through sexual contact [18]. According to the CDC, approximately 10% of HIV-positive patients can have HBV. HIV–HBV-co-infected individuals may experience worse outcomes compared to mono-infected individuals. Higher HBV viremia levels, a greater likelihood of progression to chronic HBV infection, and an increased risk of cirrhosis and hepatocellular carcinoma are also possible in this regard. A study conducted in the state of Kaduna in Nigeria included 200 people who had an HIV infection. These patients (143 women and 57 men) were screened for HBsAg markers. According to the study, 17 patients had positive results for HBsAg (8.5%). The rate for men (14.0%) was found to be higher than for women (6.3%) [19]. In the present study, positivity for HBsAg was detected in 10 (3.9%) patients. In support of this study, the rate for men (3.35%) was found to be higher than for women (0.95%). We also think that the fact that our patients were in the treatment process affected our results. High Hepatitis B rates for surface antigen (HBsAg) negativity were reported immediately after the initiation of HBV active ART in HIV–HBV-co-infected patients. HBsAg negativity was reported as ranging from 2.8 to 23% between 2012 and 2016. The data published in 2018–2019 showed that these rates remained quite stable (3.0–13.9%) [20].

Hepatitis C is among the most important infectious agents globally, and its blood-to-human transmission is one of the characteristics it shares with HIV. The infection of HIV-positive people with HCV has become the primary cause of morbidity due to liver disease all over the world in recent years. According to the CDC, approximately 21% of HIV-positive patients have HCV. An HIV/HCV co-infection leads to the more aggressive and faster progression of liver disease, an increased risk of HIV-related kidney disease, and a higher risk of cardiovascular disease and diabetes mellitus. A total of 3065 patients within the Clinical HIV Program between 2006 and 2017 in a center for HIV patients were tested for both HBV and HCV as part of a study conducted in Singapore. Although most HIV-infected people were mono-infected (86.3%), 6.0% were co-infected with HCV and 7.2% with HBV, while 0.5% had both HBV and HCV [21]. In another study conducted in Malaysia, 708 HIV-infected patients were selected, including 167 (23.6%) women and 541 (76.4%) men. Positivity was detected in 8 women (1.1%) and 122 (17.2%) men. A total of 130 patients were co-infected with HIV and HCV [22]. In our study, HIV–HCV co-infection was detected in 2/230 (0.8%), representing a low rate.

EBV is among the most common viruses in humans and is transmitted from person to person through saliva and contact with bodily secretions. It can be an important cause of cancer in people living with HIV/AIDS. Recent studies show that more than 90% of adults over 35 years of age worldwide have a seroprevalence of EBV [23]. The number of EBV-infected B cells increases in the bloodstream in most HIV-infected persons, who may develop EBV-associated opportunistic lymphomas (Burkett’s lymphoma, lymphomas that spread to large cells, and primitive cerebral lymphomas). In Burkina-Faso, EBV infection was investigated with Real-Time PCR in a group of 238 HIV-positive patients, and the rate was reported as 6.7% (16/238) [24]. In the present study, EBV DNA was investigated with Real-Time PCR, and its frequency was found to be 10/228 (4.1%).

In individuals with normal immune systems, CMV infection is asymptomatic, and latent infection develops usually after acute infection. Systemic disease with high morbidity and mortality may develop with the reactivation of a latent infection in HIV-infected patients, and serious clinical manifestations may also occur. Serious clinical conditions such as retinitis, pneumonia encephalitis, and enteritis may occur as an effect of the virus in individuals who do not receive ART. If the diagnosis of CMV is delayed in individuals with HIV, the deficiency in the immune system with the development of viremia accelerates the progression of HIV disease and causes an increase in mortality [25]. In the study of Gronborg et al. involving HIV patients, the prevalence of CMV pneumonia ranged from 20% to 60% in patients with pulmonary symptoms, while the cause of enteritis was found in 0–14% of CMV in patients with gastrointestinal symptoms. CMV retinitis was detected at a lower level, between 0 and 2.6%. In the study, CMV viremia was found to be correlated with a significantly lower CD4+ cell count and an increase in activated and apoptosis-sensitive T lymphocytes [26,27]. Zhao et al. found the overall positivity rate of CMV infection to be 29.5% in 808 HIV/AIDS patients and emphasized that a low CD4 T lymphocyte count and high HIV-1 viral load may be risk factors in these patients [28]. In our study, an HIV–CMV association was in 32/230 (13.9%) patients. The CD4 values were observed to be quite low in the CMV–HIV-co-infected patients.

*Mycobacterium tuberculosis* is a common cause of lung infections and can lead to infections in any part of the body, such as the brain, spine, and kidney. HIV and *M. tuberculosis* co-infection increases the risk of active tuberculosis. In a study conducted in Japan between 2012 and 2020, a total of 14.339 HIV-negative patients and 379 (2.5%) HIV-positive patients were identified in 14718 tuberculosis cases who underwent HIV testing. The rate of HIV-positive cases was 1.2% in women (66/5594) and 3.4% in men (313/9124) [29]. In the study of Maisa et al., opportunistic factors were found in 54 of 167 HIV-positive patients. The number of patients with tuberculosis was found to be 5.4% [30]. In the present study, the HIV–tuberculosis co-infection rate was determined as 3/228 (1.3%).

Oral candidiasis is the most common opportunistic fungal infection in patients with HIV/AIDS and is more common in the early stages of infection before ART and in the later stages of AIDS. People with a CD4+ T cell count < 200 cells/μL are at risk for this infection. The colonization of the oral mucosa with *C. albicans* and other fungi forms the basis for the development of a symptomatic infection. Erfaninejad et al. found that 113 (41%) of 276 HIV-positive patients had oral candidiasis, with *C. albicans* being the most common (64.6%) and *C. glabrata* (26.5%) the second most common in these patients [31]. Taverne-Ghadwal et al. reported that 38% of 384 HIV-positive patients had oral candidiasis, and 32% had an oral Candida colonization. The most frequently isolated species was *C. albicans* (86%) [32]. Oral candidiasis was found in one (0.4%) of 230 patients in this study, and *C. albicans* and *C. glabrata* were isolated from this patient. Esophageal candidiasis was detected in one patient (0.4%), and *C. albicans* was isolated. Also, *C. albicans* grew in the sputum of three patients and the broncholavage samples of two patients in the culture media.

A rapid decrease in CD4 T cells is evident in rapidly progressing infections after a few years. It is important to identify the underlying mechanisms and develop prognostic biomarkers and intervention strategies during this period. An untreated HIV-1 infection is characterized by an increase in CD8 cells, which is associated with the viral load level, and a decrease in CD4 target cells. When viral load levels increase, CD4 levels continue to decrease with cellular immunodeficiency, leading to AIDS and eventually mortality. Successful Antiretroviral Treatment (ART) is associated with the suppression of HIV-1 plasma RNA levels. Today’s Combined Antiretroviral Treatment (cART) is the combination of at least three different agents that consist of a non-nucleoside reverse transcriptase, an enhanced protease, or an integrase inhibitor combined with two drug nucleoside/nucleotide backbones [33].

In HIV-infected individuals, the CD4+ T cell count is an important indicator of immune function. Patients with decreased CD4+ T cell counts were associated with an increased risk of various opportunistic infections because of immunosuppression. Although it is already known that there is a direct relationship between the CD4+ T cell count and opportunistic diseases in HIV-infected individuals, different opportunistic diseases may also develop in patients with the same CD4+ T cell levels [34]. The frequency of opportunistic diseases, the agents, and the resistance characteristics of these agents differ from country to country, even between different geographical areas in the same country [35]. In the present study, a correlation was found between CD4 T cell count decline and CMV infection.

Viral load and CD4 counts are considered markers of infectivity and are important in indirectly determining whether a patient should remain in the treatment process [36]. The gender, age, race/ethnic group, and residential area categories were evaluated in a study conducted in South Carolina. Viral loads decreased more gradually during the observation period in men compared to women (higher in individuals between the ages of 13 and 19 compared to the older age group) and in HIV/AIDS patients with low CD4 counts compared to those with high CD4 counts [37]. In the present study, it was found that CD4 counts decreased in patients with an increased viral load. The highest decrease was detected in the 30–39 age group.

HIV and *T. pallidum*, in addition to sharing the same transmission routes with common risk factors, also share a synergistic interaction at the molecular level with the inflammatory process in the host. Similar to the data of our study, syphilis infections have been found to be associated with HIV, and it is known that individuals with untreated syphilis infections have significant increases in HIV viral load and significant decreases in CD4 counts. These changes have been shown to contribute to the increased risk of HIV transmission, but the causal relationship in co-infections is not clear [38,39].

## 5. Conclusions

In conclusion, a total of 107 opportunistic causative microorganisms were identified in 87 of 230 HIV-positive patients over one-year time intervals. *T. pallidum* was detected in 43 (18.6%) of these patients, CMV in 32 (13.9%), EBV in 9 (3.9%), HBV in 10 (4.3%), *C. albicans* in 7 (3%), *M. tuberculosis* in 3 (1.3%), HCV in 2 (0.8%), and *C. glabrata* in 1 (0.4%). Opportunistic infections mainly affect immunosuppressed patients and can be prevented with effective treatment modalities. Although it is already known that HIV patients may face different infections during their treatment, the results of the present study suggest that more attention should be paid to *T. pallidum*, CMV, EBV, and *C. albicans* agents. The routine follow-up of these infections considering the HIV viral load and CD4/CD8 counts is important for the quality of life of HIV patients.

## Figures and Tables

**Table 1:** The distribution of gender, place of residence, CD4+, CD8+, CD4/CD8 ratio, and HIV-RNA load among the HIV patients included in the study.

*Baseline Characteristics*	*n (%)*
Sex n (%)	
Female	33 (14.3)
Male	197 (85.7)
Residence n (%)	
Istanbul	202 (87.8)
Other	28 (12.2)
Age (median and IQR)	35.11 (27.45–44.9)
18–29	75
30–39	70
40–49	45
50–59	27
60–69	10
≥70	3
CD4 count at baseline (median and IQR)	429 (233.5–663.75)
0–200 cells/µL	49
201–350 cells/µL	41
351–500 cells/µL	42
501–700 cells/µL	49
>700 cells/µL	49
CD8 count at baseline (median and IQR)	861 (607.75–1245)
0–350 cells/µL	18
351–600 cells/µL	37
601–900 cells/µL	69
901–1500 cells/µL	72
>1500 cells/µL	34
CD4/CD8 ratio (median and IQR)	0.46 (0.21–0.78)
<0.3	77
∣0.3–0.5∣	49
∣0.51–0.7∣	34
∣0.71–1.0∣	34
>1.0	36
HIV RNA (median and IQR)	69,516.5 (5787.5–375,660)
≤100 (copies/mL)	35
∣101–1000∣ (copies/mL)	9
∣1001–10,000∣ (copies/mL)	21
∣10,001–100,000∣ (copies/mL)	61
>100,000 (copies/mL)	104

**Table 2:** The distribution of *T. pallidum*, Cytomegalovirus, Epstein–Barr virus, Hepatitis C virus, Hepatitis B virus, *M. tuberculosis*, and *Candida* spp. according to age groups.

Age	* T. pallidum *	CMV	EBV	HCV	HBV	* M. tuberculosis *	* C. albicans *	* C. glabrata *
18–29	17	5	3	1	1	1	1	1
30–39	14	8	-	-	2	-	2	-
40–49	10	10	1		3	1	3	-
50–59	1	8	4	1	4	1	1	-
60–69	1	1	1	-	-	-	-	-
≥70	-	-	-	-	-	-	-	-
Total	43	32	9	2	10	3	7	1

**Table 3:** The distribution of the infectious agents according to co-infection status and CD4 counts.

Agents	CD4 Counts (Cells/µL)				
	0–50	51–200	201–500	≥500	Total
* T. pallidum *			14	21	35
CMV *+ T. pallidum*	1	3	1	1	6
HBV *+ T. pallidum*	1			1	2
CMV	4	4	6	4	18
CMV + HBV		2			2
CMV + EBV		3	1		4
EBV		2	1	1	4
HCV			1		1
HBV			3	3	6
HCV *+ C. albicans + C. glabrata*	1				1
* M. tuberculosis *			1	1	2
* M. tuberculosis + C. albicans *		1			1
* C. albicans *	1	1	1		3
CMV *+ C. albicans*	1				1
CMV + EBV *+ C. albicans*	1				1

**Table 4:** The Spearman correlation of CD4, CD8, CD4/CD8 ratio, and HIV RNA viral load (low correlation: 0.1–0.29; moderate correlation: 0.30–0.49; high correlation: 0.50–1.00; negative values: negative correlations; positive values: positive correlations).

	Age	CD4	CD8	CD4/CD8	HIV RNA
Age	1.0	−0.038	−0.12	−0.28	0.19
CD4	−0.38	1.0	0.28	0.73	−0.42
CD8	−0.12	0.28	1.0	−0.31	0.04
CD4/CD8	−0.28	0.73	−0.31	1.0	−0.43
HIV RNA	0.19	−0.42	0.04	−0.43	1.0

**Table 5:** The correlation between CD4 count and HIV viral load and infection positivity.

	CD4 Count		CD4/CD8 Ratio		HIV RNA Load	
	r_s_	*p*	r_s_	*p*	r_s_	*p*
HIV RNA	−0.421	<0.001	−0.421	<0.001		
* T. pallidum *	0.135	0.041	0.103	0.121	0.216	0.001
CMV	−0.341	<0.001	−0.337	<0.001	0.225	0.001
EBV	−0.148	0.024	−0.148	0.025	0.133	0.043
HCV	−0.059	0.371	−0.042	0.523	0.044	0.509
HBV	−0.054	0.413	−0.004	0.952	−0.025	0.705
* M. tuberculosis *	−0.040	0.545	−0.039	0.557	0.029	0.660
* C. albicans *	−0.226	0.001	−0.228	<0.001	0.141	0.033
* C. glabrata *	−0.099	0.134	−0.099	0.136	0.107	0.105

r_s_: Spearman correlation value—low correlation: 0.1–0.29; moderate correlation: 0.30–0.49; high correlation: 0.50–1.00. *p* < 0.05 was considered statistically significant.

**Table 6:** The sensitivity and specificity rates of HIV viral load and CD4/CD8 ratio regarding positivity for different infections.

		AUC	*p*	95%CI	Sensitivity %	Specificity %
* T. pallidum *	CD4/CD8	0.576	0.121	0.485–0.667		
	HIVRNA	0.692	<0.001	0.622–0.763	90.7	52.2
CMV	CD4/CD8	0.558	0.091	0.489–0.686		
	HIVRNA	0.669	0.001	0.595–0.744	55.9	78.1
EBV	CD4/CD8	0.280	0.026	0.150–0.411	100	4.5
	HIVRNA	0.698	0.044	0.523–0.874	66.7	77.4
HCV	CD4/CD8	0.368	0.522	0.000–0.787		
	HIVRNA	0.636	0.508	0.172–1.000		
HBV	CD4/CD8	0.494	0.952	0.267–0.721		
	HIVRNA	0.465	0.705	0.341–0.588		
* M. tuberculosis *	CD4/CD8	0.401	0.55	0.251–0.550		
	HIVRNA	0.574	0.659	0.275–0.873		
* C. albicans *	CD4/CD8	0.117	0.001	0.040–0.194	100	1.3
	HIVRNA	0.737	0.033	0.504–0.969	71.4	80.3
* C. glabrata *	CD4/CD8	0.068	0.136	0.034–0.101		
	HIVRNA	0.969	0.105	0.947–0.992		

AUC: ROC area under the curve; 95% CI: 95% confidence interval. *p* < 0.05 was considered statistically significant.

## Data Availability

No new data were created.
